# Comparison of Team-Based Learning Over Conventional Didactic Lecture Among Second-Year MBBS Students

**DOI:** 10.7759/cureus.21792

**Published:** 2022-01-31

**Authors:** S Rajeswarie, K Praveen, Muralidhar Reddy Sangam, Vinay G, Raju R Bokan, Roonmoni Deka, Amandeep Kaur

**Affiliations:** 1 Microbiology, Al Azhar Medical College and Super Specialty Hospital, Thodupuzha, IND; 2 Anatomy, All India Institute of Medical Sciences, Guwahati, IND

**Keywords:** students’ perception, teaching-learning method, team-based learning (tbl), medical education, conventional didactic lecture (cdl)

## Abstract

Introduction

Team-based learning (TBL) is an active and structured learning process. This study was undertaken to develop interest among the students over the subject and to analyze the academic performance over routine conventional lecture method of teaching. This was a comparative study organized to know the effectiveness of TBL over conventional didactic lecture (CDL) and also to assess the student’s perception towards TBL.

Methods

This was an interventional study where second-year MBBS students took part. Students were divided into two groups and the same topic was discussed by using two different teaching-learning methods. Similar sessions were conducted with crossing over of groups for four different topics and assessments were done after each session simultaneously for both the groups and the results were analyzed.

Results

Assessment of each session has been counted and the mean marks with standard deviation have been tabulated for both groups A & B. The results were statistically significant for the TBL group. Students’ perception was also evaluated by using 5-point Likert scale for both the teaching methods, which revealed statistically significant score for TBL over CDL with the a p-value of 0.001.

Conclusion

Overall, TBL was a good teaching-learning method according to the students’ performance and perception. Interactive innovative and small-group sessions can be an effective tool to overcome the limitation of conventional method.

## Introduction

In the routine conventional didactic lecture (CDL), the facilitators/teachers are the primary source of information. To impart interest among students, the facilitator should use different types of teaching-learning methods with available technologies according to changing trends in the medical field. The learning strategies are to enhance the learning process and to engage the students to analyze and attain higher levels of domain in learning [1-5]. CDL is a one-way approach with less interaction with the students but team-based learning (TBL) is one active learning process that shoots up the individuals’ strengths by helping them to co-ordinate and indulge them in teamwork to achieve a common learning objective.

Amidst various teaching-learning methods, TBL is one of the effective teaching-learning methods where the students are provided with an interactive and innovative environment for learning along with the initiation of self-directed learning. The present generation of students prefers a more interactive, group activity, and lively environment of teaching and learning than the conventional lectures which are followed in India. We, in the responsible position, playing the role of a facilitator/teacher have to change the way of teaching and assessment methods. Moreover, the change should be according to the development in the technologies concerned with the use of audiovisual aid in the medical field which will be more effective in small-group teaching when compared to large group teaching.

Aim and objectives

This study was undertaken to analyze whether TBL is enhancing the academic performance or not. The main objectives were to study effectiveness of TBL over CDL and to assess the students’ perception toward TBL.

## Materials and methods

It was an interventional study conducted in the Department of Microbiology at Al Azhar Medical College and Super Specialty Hospital, Kerala, India. Out of 150 second-year MBBS students, 128 participated in the study among which 53 were males and 75 were females. Those students who were absent for any of the sessions and assessments were excluded from the study. The study was conducted between January 2019 and March 2019.

Ethical clearance

Approval from Institutional Research Board (IRB) and Institutional Ethical Committee (IEC) was obtained prior to the start of the study. Institutional Ethical clearance was obtained on 08-02-2019 and the IEC Ref. No. AAMC/IEC/2018-19/7.

Procedure and data collection

Students were randomly divided into two groups as Group A and Group B with the help of software using their roll numbers. Each group had 64 students. Informed consent was obtained from the study subjects. The same topic was assigned for both the groups. Faculty members were sensitized before the commencement of the study.

Standardization with respect to faculties regarding the topics was done before the start of the session. For Group A, the topic was discussed in form of CDL, and Group B students were subdivided into three sub-groups and they underwent TBL in the form of small-group discussion.

For Group B, topic sensitization was done in the form of hand-outs and book chapters two days prior to the session and discussion was done in form of case scenarios by three different faculties simultaneously. Individual readiness assessment test (IRAT) and group readiness assessment test (GRAT) were conducted during the discussion.

Four sessions were conducted with reversal of groups for TBL and CDL. Following each session, assessment was done by conducting one-word questions on the same day for both the groups simultaneously to assess the outcome and the results were analyzed. Furthermore, the perception of students toward TBL was also assessed by a questionnaire using a 5-point Likert scale.

Validity and reliability of questionnaire

The content validity and reliability were measured by a group of experts including an expert from the subject concerned following which Cronbach’s α value was 0.78.

Statistical analysis

Continuous variables were expressed as mean ± SD. Unpaired t-test was used to compare the marks between TBL and CDL. p-value ≤ 0.05 was considered as statistically significant. SPSS 16.0 software (IBM Corp., Armonk, New York, USA) was used for all statistical analyses.

## Results

The study was conducted over a period of three months in the department of microbiology. About 128 students participated in the study. The results thus obtained were analyzed and comparison was done between the two teaching-learning methods for four topics (Table 1).

**Table 1:** Comparison between the two teaching-learning methods for four topics

Topic No.	Conventional didactic lecture marks (Mean ± SD)	Team-based learning marks (Mean ± SD)	p-value
1	8.10±2.243	9.34±0.576	0.001
2	8.21±1.338	9.02±1.237	0.001
3	6.41±2.334	7.30±1.311	0.001
4	6.35±2.764	8.14±2.046	0.001

**Figure 1:**
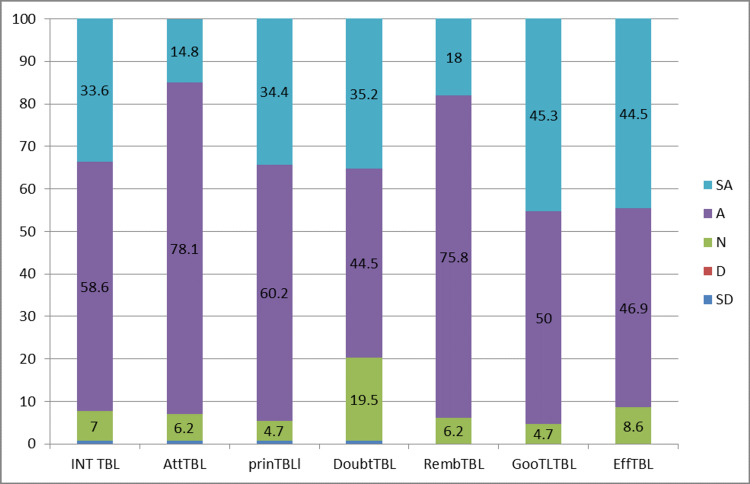
Students’ perception of TBL—5-point Likert scoring TBL: team-based learning; INT: interest; Att: attention; prin: principle; Doubt: doubt clearance; Remb: remembering; Goo: good; Eff: effective; SA: strongly agree; A: agree; N: neutral; D: disagree; SD: strongly disagree

**Figure 2:**
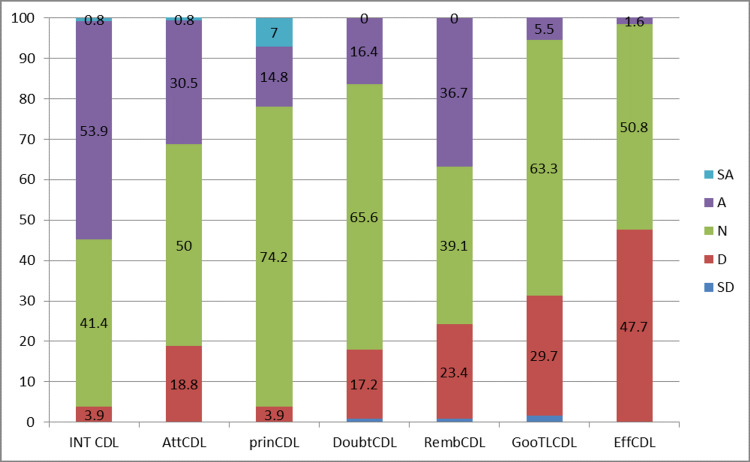
Students’ perception of CDL—5-point Likert scoring CDL: conventional didactic lecture; INT: interest; Att: attention; prin: principle; Doubt: doubt clearance; Remb: remembering; Goo: Good; Eff: effective; SA: strongly agree; A: agree; N: neutral; D: disagree; SD: strongly disagree

In all four sessions, academic performance of the students was better for those who had undergone TBL compared to those who had undergone CDL. The p-value was also statistically significant for the TBL type of teaching. Students’ perception was also evaluated following analysis of the questionnaire which was graded using 5-point Likert scale (Figures 1-2) and results were noted.

The mean rank and p-value of students’ perception for both TBL and CDL are shown in Table 2. Most of the students preferred TBL as it helped them in better understanding of the subject, to clear doubts during the session, and better retaining of acquired knowledge. 5-point Likert scale was used to obtain the feedback about the teaching-learning methods since it is categorical data, Mann-Whitney test was used to arrive at the mean rank. The feedback questionnaire is provided in the Appendices.

**Table 2:** Students’ perception about team-based learning and conventional didactic lecture TBL: team-based learning; CDL: conventional didactic lecture

No.	Questionnaire	Mean Rank		p-value
		TBL	CDL	
1.	This method was able to generate interest in the subject	163.75	93.25	0.001
2.	Attention span was good during this teaching method	170.91	86.09	0.001
3.	This method was able to understand the principles of topic	175.29	81.71	0.001
4.	This method was able to clear your doubts about the topic	174.52	82.48	0.001
5.	Will remember the topic better with this teaching method	170.19	86.81	0.001
6.	Will recommend this method as a good teaching-learning method	188.52	68.48	0.001
7.	Overall this teaching method was effective and beneficial to me	189.07	67.93	0.001

The p-value was also statistically significant for the TBL with respect to students’ perceptions. The interactive learning atmosphere during the session developed more interest among the students toward the subject rather than the routine monotonous one-way lecture.

## Discussion

The main objectives of this study were to assess the effectiveness of TBL in enhancing the academic performance over CDL and to assess the students’ perception toward TBL. The purpose of this study is to highlight the present scenario of the implementation of competency-based medical education (CBME) and changing trends in teaching-learning methods in the medical field. Besides, this study was done to assess the academic performance of the students by using students’ centered methods.

Teaching and learning is a two-way process. Learning may not take place without effective teaching. Teaching is an ever-evolving process in the medical field and it should be modernized according to the generations. Usually, it is a difficult task for the teacher who has to deliver a large amount of information in a very short stipulated time. At the same time, students should also retain or remember what was taught, interpret, and apply it as and when required.

In the present study, students who underwent TBL performed well compared to the students who were taught by the CDL. There was a significant difference between the two groups following statistical analysis.

Implementing TBL is not an easy task with the existing faculty strength, since it requires more time and manpower to utilize its complete benefit. But still, the course of the study has to be continued further compared with different types of teaching-learning methods for a longer duration to assess the long-term outcome. TBL was introduced in the medical field to improvise the teaching and to provide an active learning experience for the students, besides the concept of self-directed learning and teamwork. Qualitative analysis of students was done by open-ended questions, and results were in favor of TBL compared to lecture. Similar results were observed by some authors that academic performance improved a lot in those students who had undergone TBL and students preferred small-group teaching rather than the conventional method [2-5].

Effective teaching can occur only in presence of learning and teaching, without learning it is considered as conversation and it would be a big hurdle for the teacher to deliver more information in a little time frame [6]. Few works of literature reveal that in conventional methods of teaching, the worst affected are the below-average students [7]. Learner’s centered, small-group teaching in the form of problem-based and TBL are widely been practiced in most of the European countries which improve the students’ critical thinking and make them learn effectively [8].

In team teaching, both the learner and the facilitator are mutually benefitted. It helps the student to discuss and acquire knowledge about common and complex case scenarios which improve their performance. Students are interested to have a new mode of learning environment. Peer review, feedback, and reflections from students, build up the teachers’ interprofessional relationships and promote refinement in their field as well [9-17].

TBL also throws light over self-directed learning to encourage and motivate the student’s community. In view of generating interest toward subjects, few students preferred the conventional method over newer methods. The attention span and clearing of doubts were better dealt with in TBL sessions than in the lecture. It was also noted that students’ perception of understanding the principles of topics and retaining capacity was significantly high in TBL. Overall results of the present study reveal that majority of the students preferred and appreciated TBL as an effective teaching-learning method.

Limitations of the study

First, conducting this study as a part of a regular schedule was difficult due to time constraints. Second, all topics of the subject could not be approached in the same manner; and third: faculty strength was also a limiting factor for such an effective approach.

Recommendation

Interactive and innovative teaching methods should be introduced to ameliorate the learning process of the Indian Medical Graduates. To achieve the above goal, the faculty strength of the medical colleges should be increased.

## Conclusions

Traditional teaching methods have been still followed in many institutions which are usually teacher-centered where learning occurs in a passive manner. In the present study, second-year MBBS students inclined more toward TBL than the CDL, which was evident from their assessment scores and their perception. Novel methods of teaching should be facilitated among the students’ community which will increase their attentiveness and better retention capacity during the class hours.

## Appendices

Feedback questionnaire

Name:                                                                     Age:                      Sex:                      Roll no:

Put a tick mark in the respective column.

**Table 3:** Feedback questionnaire *TBL: team-based learning

No.	Questions	Strongly agree (5)	Agree (4)	Neutral (3)	Disagree (2)	Strongly disagree (1)
1.	TBL^*^ was able to generate interest in the subject					
2.	Attention span of students was good during the TBL^*^ session					
3.	Was able to understand the principles of topic in TBL^*^					
4.	Were able to clear your doubts about the topic in TBL^*^					
5.	Will remember the topic better with TBL^*^ teaching method					
6.	Will recommend TBL^*^ as a good teaching-learning method					
7.	Overall TBL^*^ teaching method was effective and beneficial to me					
8.	Lecture was able to generate interest in the subject					
9.	Attention span was good during the lecture session					
10.	Was able to understand the principles of topic in lecture					
11.	Were able to clear your doubts about the topic in lecture					
12.	Will remember the topic better with lecture as a teaching method					
13.	Will recommend lecture as a good teaching-learning method					
14.	Overall lecture as teaching method was effective and beneficial to me					
